# Evaluation of algorithms for photon depth of interaction estimation for the TRIMAGE PET component

**DOI:** 10.1186/2197-7364-2-S1-A13

**Published:** 2015-05-18

**Authors:** Niccolo Camarlinghi, Nicola Belcari, Piergiorgio Cerello, Giancarlo Sportelli, Francesco Pennazio, Emanuele Zaccario, Alberto Del Guerra

**Affiliations:** University of Pisa, Italy; University of Torino, Italy

The TRIMAGE consortium aims to develop a multimodal PET/MR/EEG brain scanner dedicated to the early diagnosis of schizophrenia and other mental health disorders. The PET component features a full ring made of 18 detectors, each one consisting of twelve 8x8 Silicon PhotoMultipliers (SiPMs) tiles coupled to two segmented LYSO crystal matrices with staggered layers. In each module, the crystals belonging to the bottom layer are coupled one to one to the SiPMs, while each crystal of the top layer is coupled to four crystals of the bottom layer. This configuration allows to increase the crystal thickness while reducing the depth of interaction uncertainty, as photons interacting in different layers are expected to produce different light patterns on the SiPMs. The PET scanner will implement the pixel/layer identification on a front-end FPGA. This will allow increasing the effective bandwidth, setting at the same time restrictions on the complexity of the algorithms to be implemented. In this work two algorithms whose implementation is feasible directly on an FPGA are presented and evaluated. The first algorithm implements a method based on adaptive thresholding, while the other uses a linear Support Vector Machine (SVM) trained to distinguish the light pattern coming from two different layers. The validation of the algorithm performance is carried out by using simulated data generated with the GAMOS Monte Carlo. The obtained results show that the achieved accuracy in layer and pixel identification is above the 90% for both the proposed approaches.

